# Phylogenetic Relatedness Among Plasmids Harbored by *Campylobacter jejuni* and *Campylobacter coli* Isolated From Retail Meats

**DOI:** 10.3389/fmicb.2018.02167

**Published:** 2018-09-12

**Authors:** Daya Marasini, Anand B. Karki, Mark A. Buchheim, Mohamed K. Fakhr

**Affiliations:** Department of Biological Science, The University of Tulsa, Tulsa, OK, United States

**Keywords:** *Campylobacter*, plasmids, phylogenetic relatedness, retail meats, next generation sequencing

## Abstract

*Campylobacter jejuni* and *Campylobacter coli* are two of the major causes of foodborne illness. In this study, 29 plasmids isolated from 20 retail meat isolates of *Campylobacter jejuni* and *Campylobacter coli* were fully-sequenced individually or as a part of a whole genome sequencing approach. The fully-sequenced plasmids ranged in size from 3 to 119 kb. Molecular characterization of the sequenced plasmids was based on pangenomic analysis and types of genes present on these plasmids and similar ones from GenBank. The plasmids were categorized into four different groups. These groups include type-1 that consisted mainly of pTet plasmids with the *tetO* gene, type-2 plasmids commonly found in *C. coli* strains, type-3 which has pVir plasmids, and type-4 that consisted mainly of smaller plasmids. The type-2 plasmids were unique, common among *C. coli* strains, and carried several conjugative transfer genes. The type-2 plasmids were most similar to a plasmid from *Helicobacter pullorum*. Maximum parsimony analysis and NeighborNet analysis were used to assess the phylogenetic relatedness among the 29 plasmid sequences presented in this study in addition to the other 104 plasmid sequences of *Campylobacter* species available in GenBank to date. Results from MP analysis revealed multiple lineages among *Campylobacter* plasmids which was supported by NeighborNet analysis. Clustering of plasmids did not conform to species-specific clades which suggested an intra-species dissemination of plasmids among *Campylobacter* species. To our knowledge, this is the first extensive phylogenetic analysis of *Campylobacter* plasmids sequenced to date.

## Introduction

Foodborne bacterial illness caused by *Campylobacter* spp. in the United States ranks third after *Salmonella* spp. and *Clostridium perfringens* (Scallan et al., 2011). Most of the foodborne illnesses associated with *Campylobacter* spp. have been related to *Campylobacter jejuni*; *w*hereas, the remaining have been attributed to *Campylobacter coli* (Acheson and Allos, 2001; Gillespie et al., 2002). Species from genus *Campylobacter* are known to have gained various types of antibiotic resistance, mostly tetracycline and aminoglycosides, followed by chloramphenicol (Taylor, 1986; Tenover et al., 1992). The majority of antibiotic resistance in bacteria is associated with plasmids. While several studies on plasmids of other foodborne pathogens like *Escherichia coli* and *Salmonella* spp. are available (Williams et al., 2013), only a few reports focused on plasmids of *C. jejuni* and *C. coli*. Various types of plasmids and their incompatibility groups were reported in other foodborne pathogens, but little is known about *Campylobacter* plasmids. The replicon typing and the RFLP analysis of the reference collection of ECOR, SARA, SARB and SARC (*E. coli* and *Salmonella*) plasmids showed unique RFLP patterns indicating variation among the plasmids of size greater than 30 kb (Williams et al., 2013). The IncX group of plasmids, which encode Type IV fimbriae in the *Enterobactericeae*, has also been expanded to four subtypes according to comparisons based on phylogenetic analysis (Johnson et al., 2012). Of the few plasmids studied in *C. jejuni* a majority (53%) had the tetracycline resistance gene, *tetO* (Schmidt-Ott et al., 2005). Approximately 29% of *C. jejuni* isolates obtained from bloody diarrhea samples contained plasmids that conferred tetracycline resistance (pTet) as well as virulence (pVir) plasmids (Schmidt-Ott et al., 2005). Next Generation Sequencing (NGS) technology has led to the characterization of a number of megaplasmids (up to 180.5 kb) of *C. jejuni* and *C. coli* isolated from various sources and bearing a spectrum of interesting genes such as the type VI secretion system(Gunther et al., 2016; Marasini, 2016; Marasini and Fakhr, 2016a,b,c, 2017a,b,c).

The *tetO* gene present in the most frequently encountered pTet plasmids was likely acting to maintain *Campylobacter* plasticity (Friis et al., 2007). Nucleotide sequence comparison of two tetracycline resistant plasmids of 45.2 and 44.7 kb in size showed the presence of the *tetO* gene, methylase and various homologous hypothetical genes present in both plasmids (Friis et al., 2007). These plasmids also contained various replication-associated and conjugation-associated genes that showed homology with a plasmid from *Actinobacillus actinomycetemcomitans* (Batchelor et al., 2004). The tetracycline resistance gene *tetO* present in the *C. jejuni* plasmid of 45 kb in size showed a significant similarity to the *tetM* tetracycline resistance gene of *Streptococcus* spp., indicating the possible interchange of genetic information between these bacteria (Taylor, 1986). In most of the studies, *tetO* has been found to be located on plasmids; whereas, in other studies it was chromosomally located in both *C. jejuni* and *C. coli* (Pratt and Korolik, 2005; Marasini and Fakhr, 2016a,b,c, 2017a,b,c). The *tetO* determinant was found in the chromosome as a part of a transposon gene cassette in isolates of *C. coli* derived from turkey and swine (Pratt and Korolik, 2005). Some of the pTet plasmids are also known to contain the aminoglycoside phosphotransferase gene aphA-3 and aphA-7 kanamycin resistant determinants (Tenover et al., 1992; Crespo et al., 2016; Marasini and Fakhr, 2016b). Studies of *C. jejuni* and *C. coli* showed that the aphA-7 gene was also present in the smaller plasmids of 11.5 and 9.2 kb in size (Tenover et al., 1992). The *C. jejuni* strain 81-176 was found to contain a pVir plasmid encoding genes homologous to type IV secretion system found in *Helicobacter pylori* (Bacon et al., 2000). This pVir plasmid was thought to be associated with bloody diarrhea but the connection could not be confirmed (Louwen et al., 2006).

Besides these two major types of plasmids (pTet and pVir), various other cryptic plasmids have been identified and fully sequenced (Jesse et al., 2006; Miller et al., 2007). The plasmid pTIW96 from a wild bird isolate of *C. jejuni* was 3,860 bp in size with 5 ORFs. Two of the ORFs of this plasmid were similar to pCC2228-2 (Hiett et al., 2013) found in another *C. coli* plasmid. Sequence analysis of the two cryptic plasmids of an agricultural isolate of *C. coli* showed one of the plasmids contained an ORF with homology to a plasmid from *C. upsaliensis* (Jesse et al., 2006). To date, 127 *Campylobacter* plasmids have been completely sequenced and deposited in GenBank. Most of these are small plasmids that were isolated from clinical sources. The current study aimed to determine the DNA sequences and provide a molecular characterization of numerous plasmids from *C. jejuni* and *C. coli* strains isolated from retail meat sources. Phylogenetic relatedness among 29 different *Campylobacter* plasmids, ranging from 3 to 119 kb, and those available in the GenBank were also investigated to identify possible plasmid lineages and origins.

## Materials and methods

### *Campylobacter* strains used for plasmid isolation

A total of 29 plasmids from *Campylobacter jejuni* (19) and *Campylobacter coli* (10) were characterized (Table 1). However, 23 of these plasmid sequences were previously announced as part of whole genome sequences of *Campylobacter* strains (Marasini and Fakhr, 2016a,b,c, 2017a,b,c), and the remaining 6 plasmids were fully sequenced in this study and deposited in GenBank (Table 1). These plasmids were harbored by 20 *Campylobacter* isolates previously isolated from various retail meat samples in Tulsa Oklahoma (Noormohamed and Fakhr, 2012, 2013, 2014). The selection of the bacterial isolates for this plasmid study was based on the restriction pattern analysis and PFGE screening of megaplasmids detected in a previous study (Marasini and Fakhr, 2014).

**Table 1:** Details of the plasmids used in this study which were isolated and sequenced in our laboratory.

**Name of the plasmid**	**Species/Strains**	**Source**	**Size bp**	**No. of contigs./ N50 value**	**Coverages (Average)**	**G+C**	**ORF**	**Accession no**.
pCCDM219S^*^	*C. coli* VS1219	Chicken	3,002	1	48280.83	31.7	2	MH634991
pCCDM18S^*^	*C. coli* P118	Chicken	3,304	1	3,838.10	31.4	4	MH634988
pCCDM223S^*^	*C. coli* YV1223	Pork	4,118	1	11,311.46	28	4	MH63499
pCCDM18S1^*^	*C. coli* P118	Chicken	4,374	1	5,080.96	30.8	6	MH634989
pCCDM116S	*C. coli* MG1116	Chicken liver	24,874	1	3,005.95	29.4	31	CP017870
pCCDM33	*C. coli* WA333	Chicken liver	25,058	1	1,129.32	29.3	30	CP017874
pCCDM105S	*C. coli* YF2105	Chicken liver	25,284	1	448.22	29.2	31	CP017867
pCCDM108S	*C. coli* BG2108	Chicken liver	25,286	1	618.04	29.2	31	CP017880
pCCDM140S^*^	*C. coli* XK3140	Chicken liver	26,812	1	214.67	29.3	32	MH634990
pCCDM18M^*^	*C. coli* P118	Chicken	26,824	1	188.04	29.8	33	MH634987
pCCDM224S	*C. coli* ZVI224	Pork	32,270	1	166.9	29.2	40	CP017877
pccdm1	*C. coli* HC248	Beef liver	44,064	1	917.82	28.8	49	CP013035
pccdm3	*C. coli* CO2160	Beef liver	44,228	1	343.91	27.8	47	CP013033
pccdm2	*C. coli* CE27*5*	Beef liver	44,233	1	467.76	27.9	47	CP013037
pCCDM116L	*C. coli* MG1116	Chicken liver	45,633	1	4,269.27	29	53	CP017869
pCCDM108L	*C. coli* BG210*8*	Chicken liver	46,186	1	1,272.92	28.9	55	CP017879
pCCDM105L	*C. coli* YF2105	Chicken liver	46,193	1	676.63	28.9	56	CP017866
pCCDM183	*C. coli* BP3183	Chicken liver	55,122	1	168.90	31.6	66	CP017872
pCCDM224L	*C. coli* ZVI224	Pork	55,234	1	382.86	28.3	62	CP017876
pCJDM210S	*C. jejuni* YQ2210	Turkey	5,170	1	6,305.66	32.3	7	CP017858
pCJDM100	*C. jejuni* IF1100	Chicken liver	5,209	1	5,946.27	28.7	6	CP017864
pCJDM204S	*C. jejuni* ZP3204	Chicken gizzard	5,257	1	9,982.72	32.1	9	CP017855
pCJDM67S	*C. jejuni* OD267	Chicken liver	36,602	1	754.11	26.1	49	CP014746
pCJDM218	*C. jejuni* TS1218	Chicken	43,077	1	517.56	29	48	CP017861
pCJDM204L	*C. jejuni* ZP3204	Chicken gizzard	44,436	1	276.12	28	49	CP017854
pCJDM210L	*C. jejuni* YQ2210	Turkey	44,808	1	366	28	50	CP017857
pcjDM	*C. jejuni* T121	Chicken	82,732	1	486.08	29.8	113	CP013117
pCJDM67L	*C. jejuni* OD267	Chicken liver	116,883	5 (N50 = 26245bp)	200	26.9	125	CP014745
pCJDM202 L	*C. jejuni* WP2202	Chicken gizzard	119,543	5 (N50 = 26237bp)	275	27.2	136	CP014743

* *Plasmids sequenced in this study*.

### Plasmid isolation and sequencing

Whole genomic DNA and plasmid isolation from *Campylobacter* strains, sequencing in Illumina Miseq platform and sequence assembly process in CLC workbench version 7.5.1 have been described previously (Marasini and Fakhr, 2016a,b,c, 2017a,b,c). Briefly, whole genome DNA isolation was carried out according to manufacturer's protocol with DNeasy Blood and Tissue kit (Qiagen Inc, Valencia, CA, United States) from cells grown micro-aerobically for 72 h in Mueller Hinton (MH) broth with 5% blood at 42°C. The Qiagen plasmid midi kit (Qiagen Inc, Valencia, CA, United States) was used for plasmid isolation according to manufacturer's protocol. DNA quantification was done with a Qubit 2.0 fluorimeter using high sensitivity ds DNA assay kit (Life Technologies, CA, United States) and library preparation for sequencing was completed using a Nextera XT sample preparation kit (Illumina Inc, CA, United States) as per manufacturer's instructions. Sequencing was done on Illumina MiSeq platform using Illumina MiSeq V2 reagent kit 2 × 150 cycles (Illumina Inc, CA, United States). Sequence assembly was performed using CLC Genomics Workbench version 7.5.1. Plasmid sequences with several contigs were joined and made into a single contig using contig vs. contig alignment. Joints for the contigs were confirmed by PCR and Sanger sequencing.

Plasmid sequences have been deposited in Genbank (Table 1), and announced briefly as part of whole genome sequences (Marasini and Fakhr, 2016a,b,c, 2017a,b,c). Details of all plasmids isolated from our laboratory including their Genbank accession number, number of contigs, N50 and coverages are listed in Table 1. All plasmid sequences submitted to GenBank were annotated by the NCBI Prokaryotic Genome Annotation Pipeline. The RAST online tool (http://rast.nmpdr.org/rast.cgi) (Overbeek et al., 2014) was used to annotate all plasmids evaluated in this study. Circular plasmid renderings were constructed in CLC Genomics Workbench version 7.5.1.

### Phylogenetic and genomic analysis

Pangenomic analysis of all plasmids from our laboratory was used to group plasmids in this study according to presence of different genes. Core genome and pangenome analysis for each group of plasmids from our laboratory including similar plasmids from GenBank was carried out using the GView server (https://server.gview.ca/). In addition to our 29 plasmid sequences, all available plasmid sequences of *Campylobacter* species were included to study the phylogenetic relatedness and possible transmission and origin of lineages of these plasmids. A total of 134 plasmid sequences of *Campylobacter* species (03/28/2018) from GenBank including one plasmid sequence of *Helicobacter pullorum* (plasmid 229336_12) were aligned. In GView server, blast analysis (nucleotide) was carried out using GenBank files of plasmid sequences with e-value (<1e-10), alignment length cutoff value (100) and percent identity cutoff value (80). For phylogenetic analysis, sequence alignment was done using the online version of MAFFT version 7 (https://mafft.cbrc.jp/alignment/server/) (Kuraku et al., 2013). Because the resulting alignment exhibited regions of non-overlap for various plasmids, a Maximum Parsimony (MP), character-based approach to phylogenetic analysis was used (i.e., neither distance-matrix methods nor nucleotide substitution models can be applied when extensive non-overlap exists). PAUP (Swofford, 2002) and MEGA 6 (Tamura et al., 2013) were used for phylogenetic construction by MP. Relative branch support was assessed using the bootstrap from 1,000 replicates. For comparison and validation of phylogenetic relatedness of plasmids inferred from MP tree, we also performed NeighborNet analysis with SplitsTree4 (Huson and Bryant, 2006).

## Results

### Molecular charcterization of the sequenced plasmids

**Figure 1:**
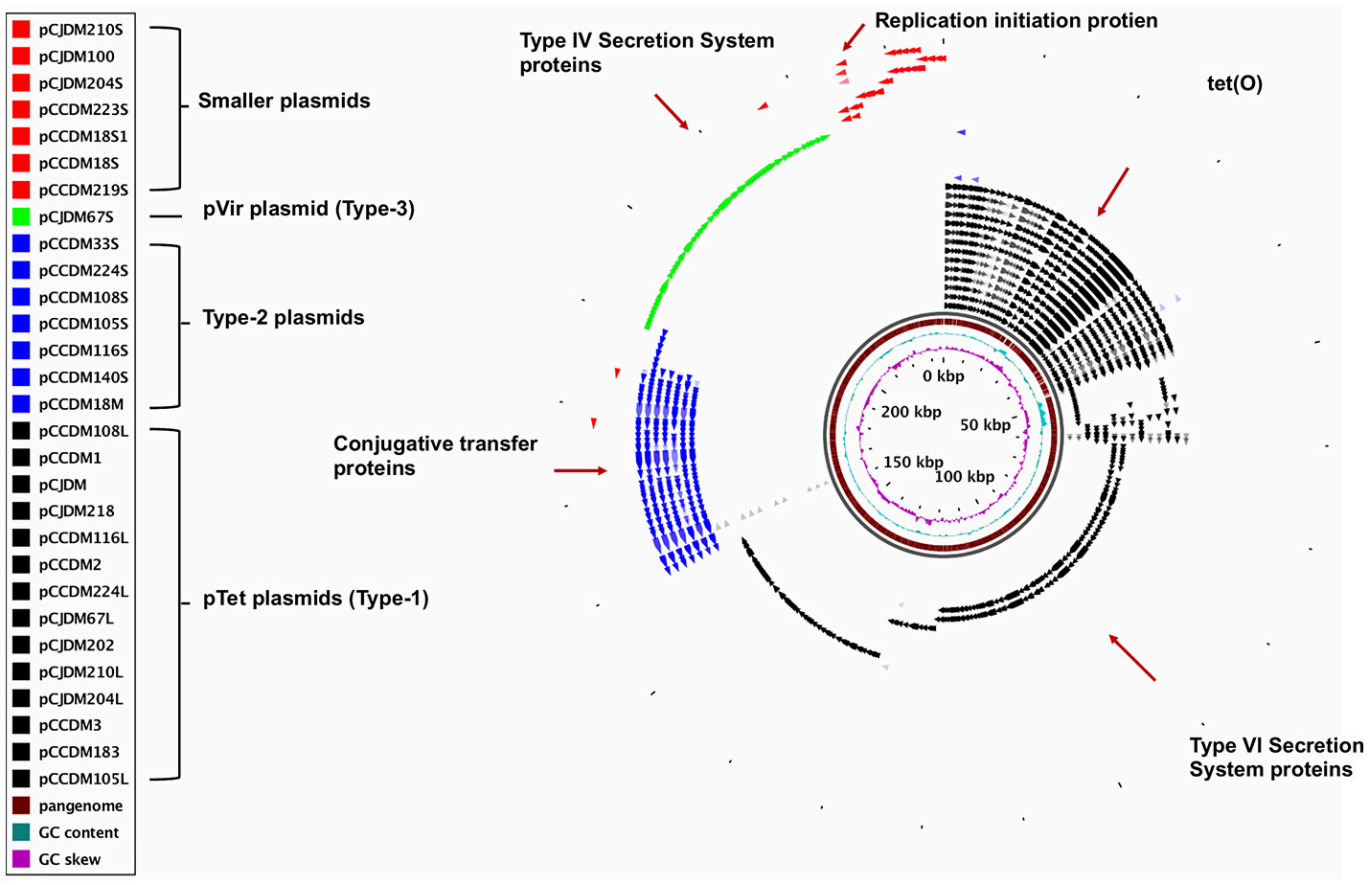
Different types of plasmids identified in our study. Circular Figure represents the pangenome analysis for all plasmid sequences from our laboratory. Individual slot (circle) in the figure represent one plasmid sequence. Pangenome analysis for all plasmid sequences was completed with GView server.

A total of 29 plasmids were fully sequenced using the Illumina MiSeq desktop sequencer. A total of 19 plasmids from *Campylobacter coli* and 10 plasmids from *C. jejuni* were sequenced. The sizes of circular plasmids ranged from 3,002 to 119,543 bp. Based on pangenomic analysis and types of genes present, we categorized plasmids into four different groups (Figure 1). These groups include (1) type-1 plasmids (pTet plasmids) with *tetO* gene, (2) type-2 plasmids commonly found in *C. coli* strains, (3) type-3 plasmids (pVir plasmids) and (4) type-4 plasmids (plasmids < 6000 bp). All plasmids from our study are listed in Table 1 and similar plasmids found in GenBank for each group after blast analysis are listed in Table 2.

**Table 2:** List of all plasmids found in *C. jejuni* and *C. coli* strains from our laboratory and GenBank used for core genome and pangenome analysis.

**Name of plasmid**	**Species/Strains**	**Source of isolation**	**Size (bp)**	**Accession number**	**Type of plasmid**
pccdm1 ^*^	*C. coli* HC2-48	Beef liver	44,064	CP013035	1
Pccdm3 ^*^	*C. coli* CO2-160	Beef liver	44,228	CP013033	1
Pccdm2 ^*^	*C. coli* CF2-75	Beef liver	44,233	CP013037	1
pCC31	*C. coli*	–	44,707	AY394560	1
pFB1TET	*C. coli* FB1	Human feces	44,826	CP011017	1
pCCDM116L ^*^	*C. coli* MG1-116	Chicken liver	45,633	CP017869	1
pCCDM108L^*^	*C. coli* BG2-108	Chicken liver	46,186	CP017879	1
pCCDM105L^*^	*C. coli* YF2-105	Chicken liver	46,193	CP017866	1
pRM4661_48kbp	*C. coli* RM4461	not reported	47,962	CP007182	1
Unnamed plasmid	*C. coli* Tx40	Food borne	48,048	KX686749	1
pRM5611_48kb	*C. coli* RM5611	not reported	48,422	CP007180	1
pCCDM183 ^*^	*C. coli* BP3-183	Chicken liver	55,122	CP017872	1
pCFSAN032805_1	*C. coli* CFSAN032805	Chicken breast	55,122	CP023546	1
pN29710-1	*C. coli* CVM N29710	Retail meats	55,127	CP004067	1
pCCDM224L ^*^	*C. coli* ZV1- 224	Pork	55,234	CP017877	1
pCC14983A-1	*C. coli*	House fly	180,543	CP017026	1
pMTVDSCj16-1	*C. jejuni* MTVDSCj16	Chicken cecal content	42,686	CP017419	1
pCJDM218 ^*^	*C. jejuni* TS1-218	Chicken	43,077	CP017861	1
pTet	*C. jejuni* S3	not reported	43,222	CP001961	1
pTet	*C. jejuni* ICDCCJ07001	Clinical	44,084	CP002030	1
p11601MD	*C. jejuni* 11601MD	Turkey	44,095	KJ646012	1
pCJDM204L ^*^	*C. jejuni* ZP3-204	Chicken gizzard	44,436	CP017854	1
pMTVDSCj13-1	*C. jejuni* MTVDSCj13	Chicken cecal content	44,687	CP017418	1
pCJDM210L ^*^	*C. jejuni* YQ2-210	Turkey	44,808	CP017857	1
pMTVDSCj07-1	*C. jejuni* MTVDSCj07	Chicken cecal content	44,917	CP017416	1
pTet	*C. jejuni* 81-176	not reported	45,025	CP000549	1
pTet	*C. jejuni* 81-176	not reported	45,025	AY394561	1
pRM1246_ERRC	*C. jejuni* RM1246-ERRC	Human	45,197	CP022471	1
pTet	*C. jejuni* 81176	not reported	45,210	AY714214	1
pCJP002	*C. jejuni* YH002	Calf liver	45,904	CP020775	1
pTet-M129	*C. jejuni* M129	Clinical	46,448	CP007750	1
Unnamed plasmid	*C. jejuni* FDAARGOS_265	Clinical isolate	46,746	CP022078	1
pTet-D42a	*C. jejuni* D42a	Chicken cecum	46,761	CP007752	1
Unnamed plasmid	*C. jejuni* 00-2544	Human feces	46,902	CP006710	1
pCj1	*C. jejuni* 01-1512	Human	48,872	CP010073	1
pCJ14980A	*C. jejuni* 14980A	Turkey feces	50,689	CP017030	1
pFORC46.1	*C. jejuni* FORC_046	Human feces	51,522	CP017230	1
pCFSAN032806	*C. jejuni* CFSAN032806	Chicken breast	55,132	CP023544	1
pcjDM ^*^	*C. jejuni* T1-21	Retail chicken	82,732	CP013117	1
pCJDM67L ^*^	*C. jejuni* OD2-67	Chicken liver	116,883	CP014745	1
pCJDM202 ^*^	*C. jejuni* WP2-202	Chicken gizzard	119,543	CP014743	1
pCCDM116S ^*^	*C. coli* MG1116	Chicken liver	24,874	CP017870	2
pCFSAN032805_2	*C. coli* CFSAN032805	Chicken breast	25,046	CP023547	2
pCCDM33S ^*^	*C. coli* WA333	Chicken liver	25,058	CP017874	2
pCCDM105S ^*^	*C. coli* YF2105	Chicken liver	25,284	CP017867	2
pCCDM108S ^*^	*C. coli* BG2108	Chicken liver	25,286	CP017880	2
pCC42yr	*C. coli* 15-537360	Human	26,269	CP006703	2
pCCDM140S ^*^	*C. coli* XK3140	Chicken liver	26,812	MH634990	2
pCCDM18M ^*^	*C. coli* P118	Chicken	26,824	MH634987	2
pOR12CC42	*C. coli* OR12	Organic chicken farm	27,987	CP013736	2
pCC42	*C. coli* FB1	Human feces	29,115	CP011016	2
pCCDM224S ^*^	*C. coli* ZV1224	Pork	32,270	CP017876	2
pRM1875_35kb	*C. coli* RM1875	not reported	35,364	CP007184	3
pOR12vir	*C. coli* OR12	Organic chicken farm	37,395	CP013734	3
pCJDM67S ^*^	*C. jejuni* OD2-67	Chicken liver	36,602	CP014746	3
pCj2	*C. jejuni* 01-1512	Human	36,604	CP010074	3
pVir	*C. jejuni* IA3902	Sheep	37,174	CP001877	3
pVir	*C. jejuni* 81-176	not reported	37,468	AF226280	3
pVir	*C. jejuni* 81-176	not reported	37,473	CP000550	3
pCCDM219S ^*^	*C. coli* VS1-219	Chicken	3,002	MH634991	4
pCC14983A-3	*C. coli* 14983A	Housefly	3,142	CP017028	4
pCC2228-2	*C. coli* RM2228	not reported	3,303	DQ518171	4
pCCDM18S ^*^	*C. coli* P118	Chicken	3,304	MH634988	4
P3384	*C. coli*	not reported	3,316	AY948116	4
pRM1875_3.3kb	*C. coli* RM1875	not reported	3,324	CP007186	4
pCCT1	*C. coli*	not reported	3,327	X82079	4
pCCT2	*C. coli*	not reported	3,344	X82080	4
pRM1875_3.4kbp	*C. coli* RM1875	not reported	3,347	CP007187	4
pCCDM223S ^*^	*C. coli* YV1-223	Pork	4,118	MH634992	4
pCCDM18S1 ^*^	*C. coli* P118	Chicken	4,374	MH634989	4
pCJ01	*C. jejuni*	not reported	3,212	AF301164	4
pTIW94	*C. jejuni* S4-2	Wild bird feces	3,860	KF192842	4
pCJ419	*C. jejuni*	not reported	4,013	AY256846	4
pCJDM210S ^*^	*C. jejuni* YQ2210	Turkey	5,170	CP017858	4
pCJDM100 ^*^	*C. jejuni* IF1-100	Chicken liver	5,209	CP017864	4
pCJDM204S ^*^	*C. jejuni* ZP3-204	Chicken gizzard	5,257	CP017855	4

*The pCCDM105L, pCCDM18M, pCJDM67S, and pCJDM204S sequences were used as reference for blast analysis for type 1, type 2, type 3 and type 4 plasmids respectively. Type 1, type 2, type 3, and type 4 plasmids are highlighted in gray, blue, green, and red respectively*.

* *plasmids isolated and sequenced in our laboratory*.

### Type-1: pTet plasmids

**Figure 2:**
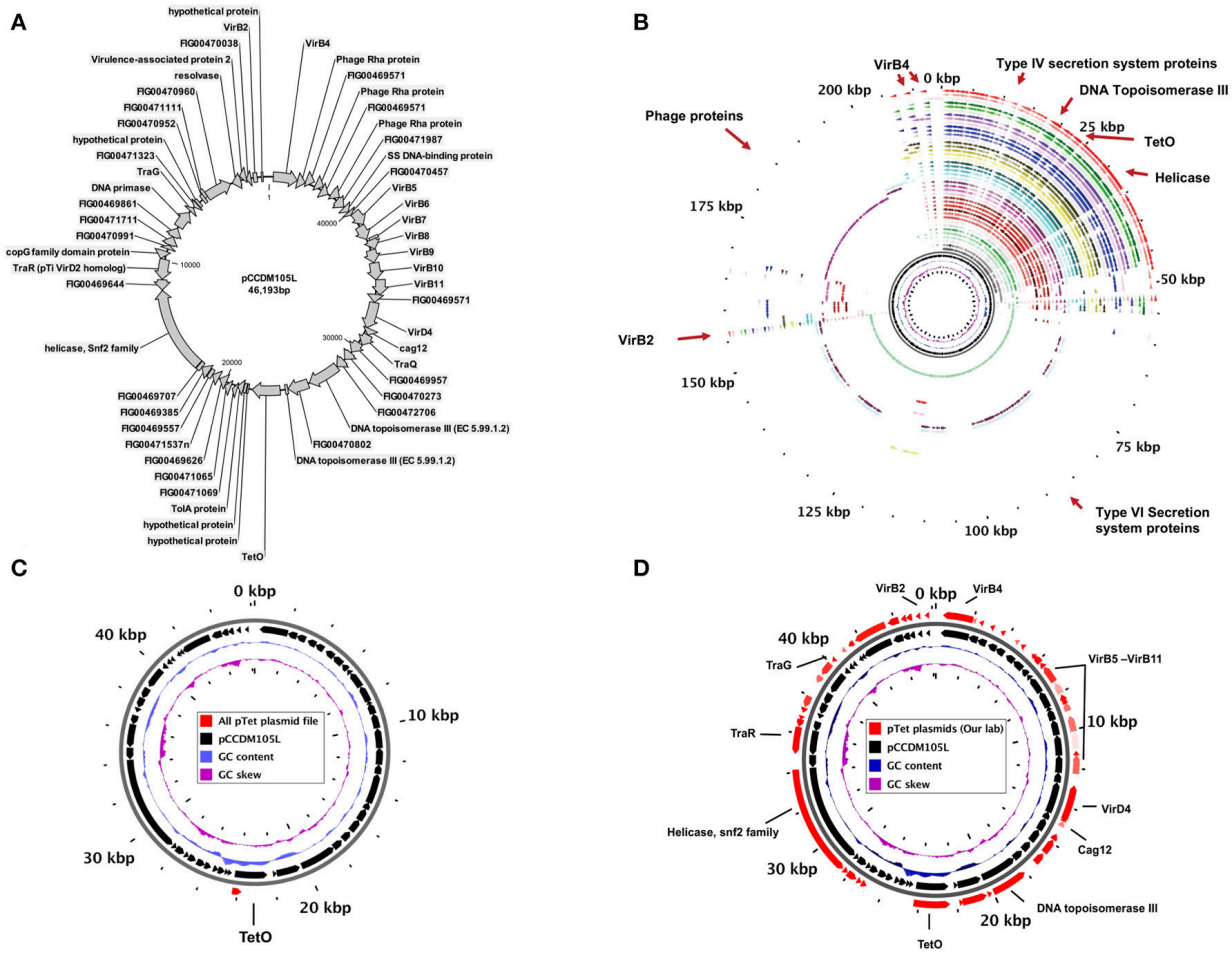
**(A)** Circular map of pTet (type-1) plasmid (pCCDM105L) showing the presence of various genes, **(B)** pangenome of pTet plasmids avialable in Genbank (incuding all pTet plasmids from our laboratory), **(C)** core genome for all pTet plasmids (red arrows in the outer circle indicate the core genome of all plasmid sequences used in the study), and **(D)** core genome among pTet plasmids isolated and sequenced from our laboratory.

The most prevalent plasmid type in *C. jejuni* and *C. coli* strains was Type 1 (pTet). Of the 29 plasmids that were isolated and sequenced, 14 were pTet plasmids (Figure 1, Table 2). Plasmid pCCDM105L served as as an examplar for all pTet plasmids and also was used as reference for core genome and pangeome analysis (Figures 2A–D). Core genome analysis among pTet plasmids isolated from our laboratory showed various genes including genes for the Type IV secretion system (virB2, virB4, virB5, virB6, virB7, virB8, virB9, virB10, and virB11 genes) as core genome (Table 3, Figure 2D, Supplementary Table 1). The core genome among pTet plasmids from our laboratory is summarized in Table 3. However, only the gene for TetO was found as core genome for all pTet plasmids of *C. jejuni* and *C. coli* from Genbank (including our 14 pTet plasmids) (Figure 2C, Supplementary Table 2). Pangenome analysis showed that most pTet plasmids share similar genomic composition and size, however, a few were determined to be megaplasmid due to the presence of extra DNA length that included some genes (Figure 2B, Supplementary Table 3). Extra Mu-like prophage genes are found to be inserted in the pcjDM plasmid (Marasini and Fakhr, 2016c), whereas, pCJDM67L, pCJDM202 (Marasini and Fakhr, 2016a) and pCC14983A-1 (from Genbank) harbor extra genes including several associated with the Type VI secretion system.

**Table 3:** The common genes (with identified functions only) present in all of type-1 (pTet) plasmids from our laboratory isolates (details and percentage identity in Supplementary Table 1).

Core genome encoding the following proteins*	**Function**
*TetO*	Tetracycline resistance protein
Helicase, snf2 family	helicase, Snf2 family
TraR	IncQ plasmid conjugative transfer DNA nicking endonuclease (pTi VirD2 homolog)
Ribbon-helix-helix protein	Ribbon-helix-helix protein, copG family domain protein
DNA primase	DNA primase (EC 2.7.7.-)
TraG	IncQ plasmid conjugative transfer protein TraG
Site-specific recombinase, resolvase family	Site-specific recombinase, resolvase family
Virulence-associated protein 2	Virulence-associated protein 2
VirB2	Major pilus subunit of type IV secretion complex
VirB4	ATPase provides energy for both assembly of type IV secretion complex and secretion of T-DNA complex
Phage Rha protein	Phage Rha protein
Single-stranded DNA-binding protein	Single-stranded DNA-binding protein
VirB5	Minor pilin of type IV secretion complex
VirB6	Inner membrane protein of type IV secretion of T-DNA complex, VirB6
VirB7	Lipoprotein of type IV secretion complex that spans outer membrane and periplasm
VirB8	Inner membrane protein forms channel for type IV secretion of T-DNA complex
VirB9	Outer membrane and periplasm component of type IV secretion of T-DNA complex, has secretin-like domain
VirB10	Inner membrane protein forms channel for type IV secretion of T-DNA complex
VirB11	ATPase required for both assembly of type IV secretion complex and secretion of T-DNA complex
VirD4	Coupling protein, ATPase required for T-DNA transfer
cag12	cag pathogenicity island protein
TraQ	IncQ plasmid conjugative transfer protein (RP4 TrbM homolog)

* *Only genes with identified functions are included, all hypothetical proteins are excluded in the list of core genome*.

Few genes responsible for virulence and antibiotic resistance were found in different plasmids (Supplementary Table 3). A gene encoding virulence-associated protein 2 (VapD) was found in all pTet plasmids from our laboratory. The protein kinase gene was present in pccdm2, pccdm3, pCJDM210L and pCJDM204L. An aminoglycoside phosphotransferase gene was present in pccdm1, pCJDM, pCCDM183, and pCCDM224L. Histidine kinase and DNA-cytosine methyltransferase were present only in pCCDM224L. Kanamycin kinase, uridine phosphorylase, spectinomycin adenyl transferase, hygromycin B-phosphorylase, pyrrolidone–carboxylase peptidase, aminoglycoside adenyltransferase and streptothiricin acetyl transferase were present in pCCDM183 (Marasini and Fakhr, 2017c). The pCJDM plasmid harbors most of the multidrug resistance genes that are also present in pCCDM183 except uridine phosphorylase and spectinomycin adenyl transferase (Marasini and Fakhr, 2016c). All genes present in pTet plasmids and percentage similarity to other plasmids are listed in Supplementary Table 3.

### Type-2: *campylobacter coli* specific plasmids

**Figure 3:**
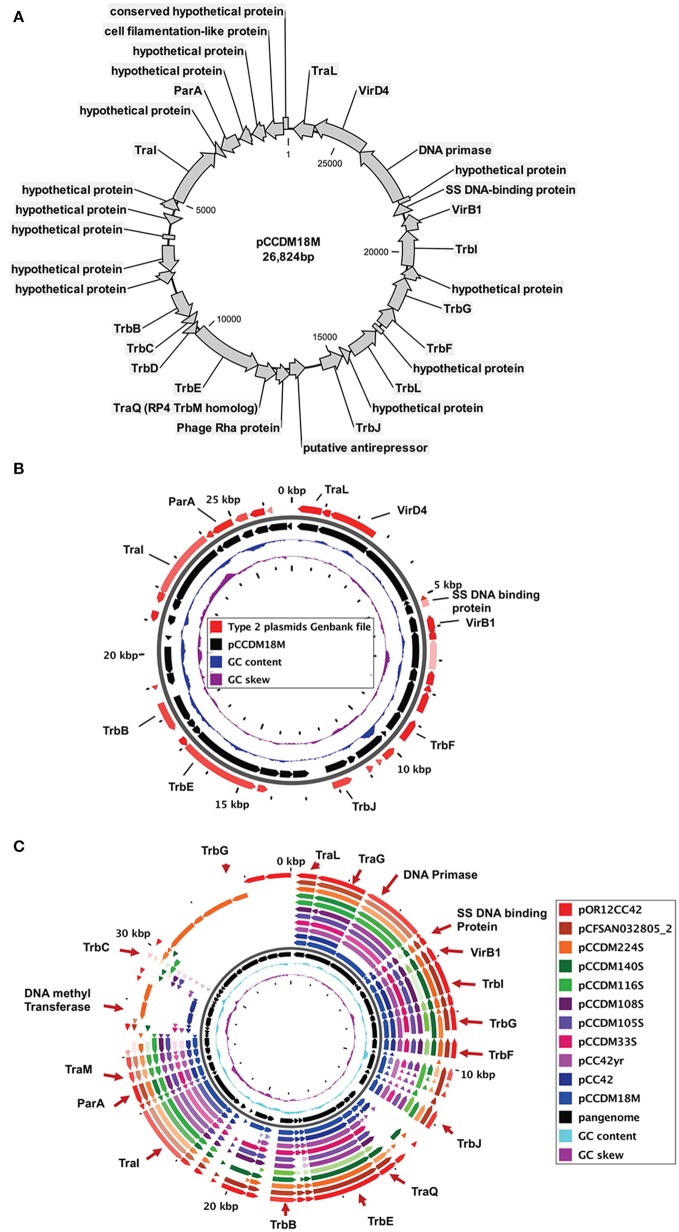
**(A)** Circular map of the type-2 plasmid (pCCDM18M), **(B)** core genome among all type-2 plasmids used in this study (Table 2, Supplementary Table 4), and **(C)** pangenome for all type-2 plasmids used in this study (Supplementary Table 5).

The type-2 plasmids are -the second-most prevalent group from our study (Figure 1, Table 2). These plasmids were found only in *C. coli* strains and were not found in any of the *C. jejuni* strains screened in our study. Type-2 plasmids range from 24 to 32 kb in size. Type-2 plasmids from our laboratory and similar plasmid sequences from GenBank are listed in Table 2. The plasmid sequence for pCCDM18M was used as reference for Blast, core genome and pangenomic analyses (Figures 3A–C, Supplementary Tables 4, 5). Results from core genome analysis among type-2 plasmids and similar plasmids from GenBank are presented in Table 4 (Figure 3B). A number of trb genes responsible for conjugative transfer were identified in these plasmids. A larger percentage of genes were conserved among the type 2 plasmids as compared to the pTet plasmids. In addition to these transfer genes, virD4, traI, gene for single-stranded DNA binding protein and traQ were common among all type-2 plasmids (Figures 3B,C, Table 4, Supplementary Tables 4, 5). Few genomic differences were found among these plasmids (Supplementary Table 5). Meanwhile, few genes related to Type IV secretion system, virB1, putative antirepresser, phage Rha proteins and mobile element protein were detected in several plasmids of this group (Supplementary Table 5).

**Table 4:** Core genome for type-2 plasmids (including all plasmids from GenBank) presented in Table 2.

Core genome encoding the following proteins*	**Function**
TraL	IncP-type DNA transfer protein
VirD4	Type IV secretion system protein VirD4
Single-stranded DNA-binding protein	Single-stranded DNA-binding protein
VirB1	Bores hole in peptidoglycan layer allowing type IV secretion complex assembly to occur
TrbI	Conjugative transfer protein TrbI
TrbG	Conjugative transfer protein
TrbF	Conjugative transfer protein
TrbL	Conjugative transfer protein
TrbJ	Conjugative transfer protein
TraQ	IncQ plasmid conjugative transfer protein (RP4 TrbM homolog)
TrbE	Conjugative transfer protein
TrbD	Conjugative transfer protein
TrbB	Conjugative transfer protein
TraI	IncP-type DNA relaxase
ParA	Chromosome (plasmid) partitioning protein
Signal peptidase	Signal peptidase I
TraM	Conjugal transfer protein
Cell filamentation-like protein	Cell filamentation-like protein

*Details and percentage identity are available in Supplementary Table 4*.

* *Only genes with identified functions are included, all hypothetical proteins are excluded in the list of core genome*.

### Type-3: pVir type of plasmid

**Figure 4:**
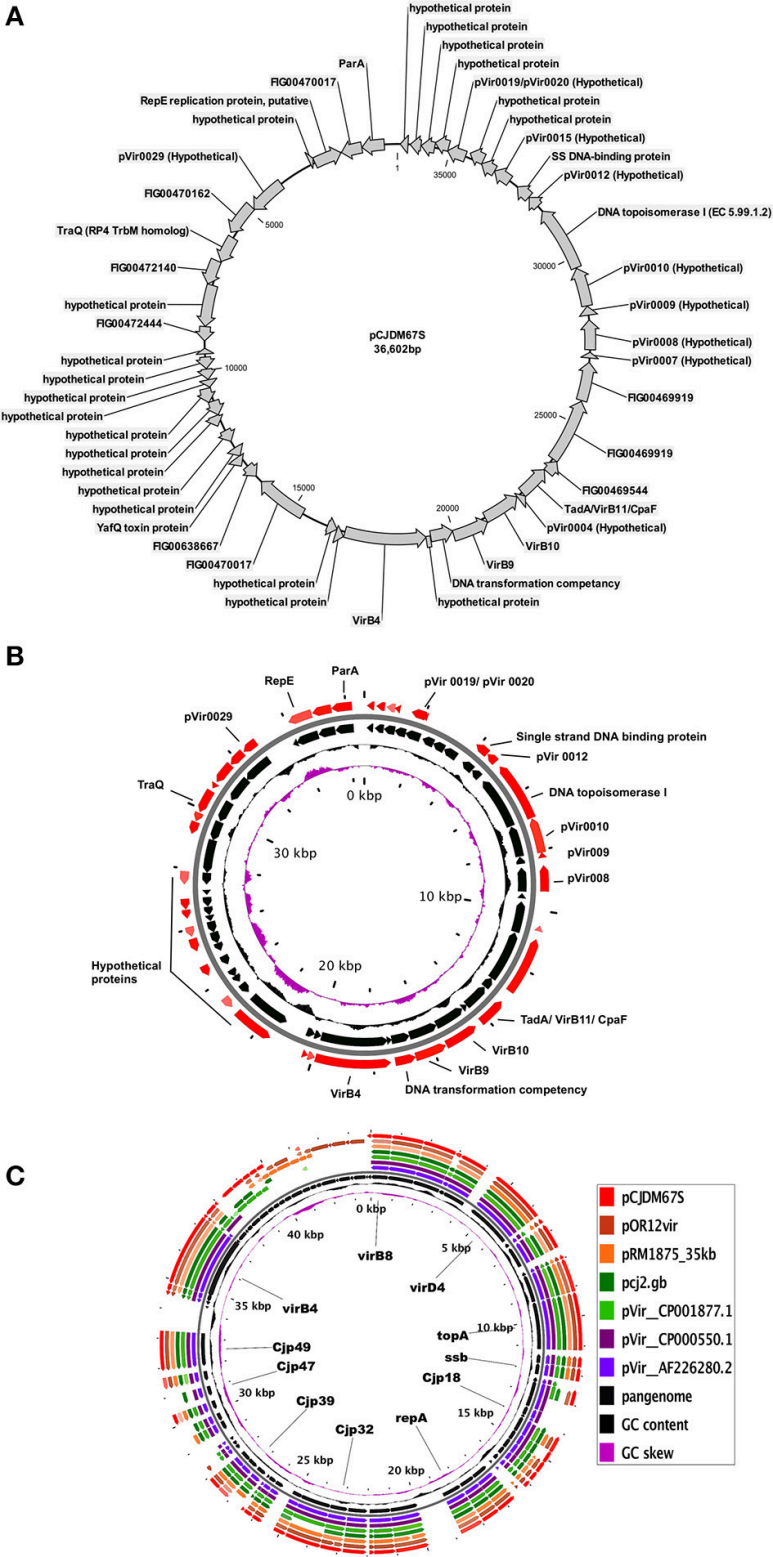
**(A)** Circular map of the plasmid, pCJDM67S, **(B)** core genome of pVir plasmids (Red arrows in outer circle represent core genome), and **(C)** pangenome of all pVir plasmids used in study.

There was only one plasmid of type-3 (pCJDM67S) among those sequenced for this project (Table 2, Figure 4A). This plasmid is similar to the pVir plasmid that was thought to be a virulence plasmid (Bacon et al., 2002). This plasmid also contains most of the hypothetical proteins observed in the pVir plasmid of *Campylobacter jejuni* 81-176 (Bacon et al., 2002) (Supplementary Tables 6, 7). The pCJDM67S plasmid shares ssb, genes for DNA topoisomerase, VirB10, VirB9, DNA transformation competancy protein, VirB4, TraQ, and RepE as core genome similar to other pVir plasmids (Table 5, Figures 4B,C). Details of all genes present among all pVir plasmids (pangenome) used in this study are presented in Supplementary Table 7.

**Table 5:** Core genome for all pVir plasmids of *C. jejuni* and *C. coli* strains used in this study (see Table 2).

Core genome encoding the following proteins*	**Functions**
Single-stranded DNA-binding protein	Single-stranded DNA-binding protein
TopA	DNA topoisomerase I (EC 5.99.1.2)
TadA/VirB11/CpaF, TadA	Type II/IV secretion system ATP hydrolase
VirB10	Type IV secretion/competence protein
VirB9	Type IV secretion/competence protein
VirB8/ DNA transformation competancy	DNA transformation competancy
VirB4	ATPase required for both assembly of type IV secretion complex and secretion of T-DNA complex
TraQ	IncQ plasmid conjugative transfer protein (RP4 TrbM homolog)
RepE	RepE replication protein, putative
ParA	Plasmid partitioning protein

* *Only genes with identified functions are included, all hypothetical proteins are excluded in the list of core genome*.

### Type-4: small plasmids

**Figure 5:**
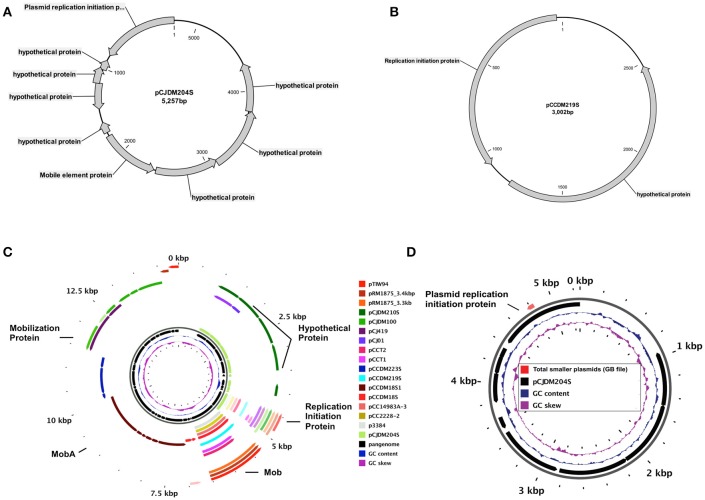
Circular map of the small (<6 kb) plasmids, **(A)** pCJDM204S, **(B)** pCCDM219S, **(C)** pangenome for all smaller plasmids used in the study and **(D)** core genome for all smaller plasmids of *C. jejuni* and *C. coli* strains used in this study from GenBank.

Seven small plasmids (<6 kb) were included in our study (Table 2, Figure 1). Except for pCJDM204S and pCJDM210S, which shared some homologous genes between them, remaining small plasmids did not share similar genetic composition. Most of these plasmids contain hypothetical protein-coding genes and replication initiater genes (Figures 5A–D). Published plasmid sequences from our laboratory and some similar plasmids from GenBank share a replication initiation protein as core genome (Figure 5D, Supplementary Table 8). However, one of these sequences, pCCDM223S, only harbors hypothetical proteins (Figure 5C, Supplementary Table 9).

### Phylogenetic analysis

**Figure 6:**
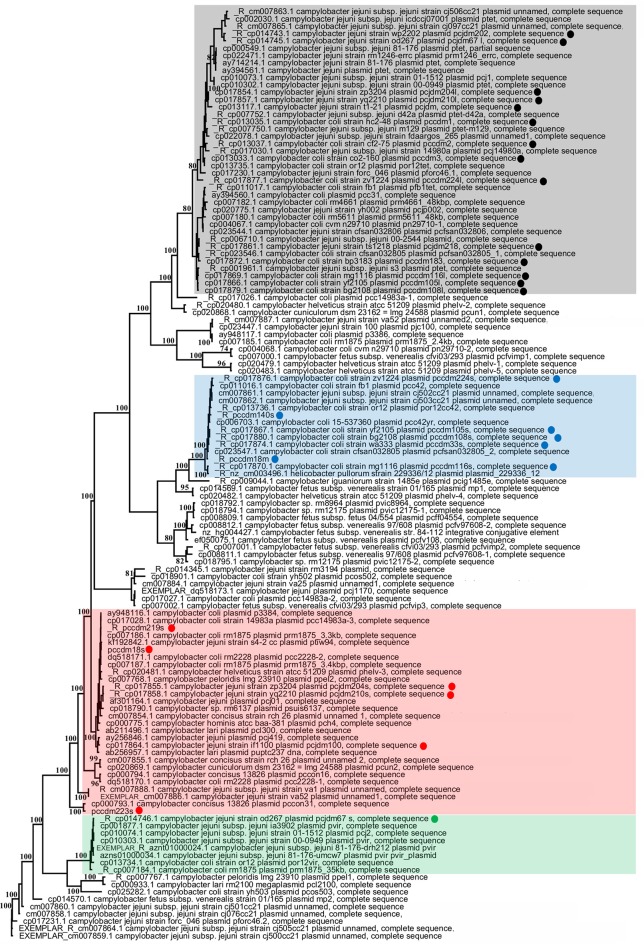
Maximum parsimony tree for all plasmid sequences of *Campylobacter* species (sequences from our laboratory are highlighted with colored circles). Categorization of plasmids from pangenomic analysis (Figure 1) are represented with shaded colors [type-1 (black), type-2 (blue), type-3 (green) and type-4 (red)] on the phylogenetic tree. Only boostrap values >70 are are mapped to the phylogenetic tree. Duplicate plasmid sequences were excluded from the analysis. The results of NeighborNet analysis for *Campylobacter* plasmids are illustrated in Supplementary Figure S1.

The original alignment comprised 134 plasmid sequences. Seven duplicate sequences were excluded for the final round of phylogenetic analyses. Results from the MP analysis are shown in the Figure 6. The MP tree supported a distinctive clade of pTet plasmids (type-1) and pVir plasmids (type-3) from all *Campylobacter* species which consisted plasmids only from *C. jejuni* and *C. coli* strains. Type-2 plasmids from *C. coli* strains in our study and other similar plasmids from Genbank also form a separate clade in phylogenetic tree. Although, most type-2 plasmids are from *C. coli* strains, two plasmids from published sequences of *C. jejuni* are also included in this group. A single plasmid from our laboratory (pcjdm67) is allied in a monophyletic group of type-3 plasmids (Figure 6). Five small plasmids (< 6 kb) from our study form part of a monophyletic group within a paraphyletic type-4 alliance (Figure 6). The pCCDM223S sequence forms a separate cluster with plasmid pCCON31 (from *C. concisus*) in the paraphyletic type-4 group (Figure 6). Numerous well-supported lineages (bootstrap values >95) are resolved by MP analysis of all plasmids from *Campylobacter* species. Not all plasmid sequences could be unambiguously categorized by pangenomic analysis (Figure 6). No species-specific clade was detected. Largely due to extensive regions of non-overlap between divergent plasmid sequences (i.e., missing data), relationships among pangenomic types and other major lineages are not resolved by these data. Results of the NeighborNet analysis revealed several major aggregates of plasmid sequences, all of which corresponded to robust branches on the MP tree (Supplementary Figure S1).

## Discussion

The literature lacks much information about plasmids of *C. jejuni* and *C. coli* despite the fact that these two organisms are major causes of foodborne illness (Scallan et al., 2011). A total of 29 plasmids of different origins and from both *C. jejuni* and *C. coli* were fully sequenced for this investigation using the Illumina MiSeq technology. After complete analysis of the annotated genes by RAST (Overbeek et al., 2014), three major groups of plasmids (type 1, 2, and 3) and a few small plasmids less than 6 kb (type 4) were identified. Other foodborne pathogens, such as *Salmonella* spp. and *E. coli*, are also known to have variable plasmids as well as various types of incompatibility groups (Johnson et al., 2012; Williams et al., 2013). Thus, the diversity among plasmids from these pathogens is not unique, but confirms the assertion that plasmid diversity from even closely-related bacteria can be immense (Taylor et al., 1983; Tenover et al., 1985).

Only the gene for tetracycline resistance (*tetO*) is found to be in core genome among all pTet plasmids (Figure 2C). As noted previously, the *tetO* gene present in pTet plasmids (type-1) also shares sequence similarity with the *tetM* gene of *Streptococcus* spp., indicating the possibility of a genetic exchange between Gram-positive and Gram-negative bacteria (Taylor, 1986). Although the *tetO* gene found in pTet plasmids from *Campylobacter* is regarded as homologous to tetracycline resistance genes from other bacteria, the genetic composition of *Campylobacter* plasmids shows little similarity to plasmids of other bacteria. The presence of the *tetO* gene in both the chromosome and the plasmids of *C. jejuni* and *C. coli* indicates that the gene was either present in the chromosome and was later transferred with the integrated plasmids, or it might have reached the chromosome following acquisition of an integrated plasmid (Pratt and Korolik, 2005; Crespo et al., 2012). The high prevalence of pTet plasmids in *Campylobacter* strains from our study is similar to previous reports of clinical isolates in Germany (Schmidt-Ott et al., 2005). The presence of pTet (type-1) and pVir (type-3) in *Campylobacter* was also discussed in the previous investigation (Schmidt-Ott et al., 2005). We concur with Schmidt-Ott et al. (2005) that pVir plasmids are less prevalent than pTet plasmids in *Campylobacter* strains.

The pTet plasmids harbor important genes responsible for conjugation and virulence, exemplified by the Type IV secretion system (Bacon et al., 2000). The Type IV secretion system was reported in chromosomes of *Helicobacter pylori* (Fernandez-Gonzalez and Backert, 2014) and was conserved in the plasmids and various genomic islands in *Campylobacter fetus* (Graaf–van Bloois et al., 2016). Many hypothetical proteins of unknown function were observed in several previously-characterized plasmids (Batchelor et al., 2004) and also in plasmids sequenced for this investigation. Some of the genes responsible for conjugation are similar to genes from *Actinobacillus actinomycetemcomitants* (Batchelor et al., 2004), which might indicate transfer of conjugative genes between nonrelated microbes. Some of the pTet plasmids (pcjDM, pCCDM183, pccdm1, and pCCDM224L) were found to possess the aminoglycoside phosphotransferase genes. These pTet plasmids are all greater than 48 kb in size except pccdm1 which is only 44 kb in size. Some plasmids (i.e., pcjDM and pCCDM183) contained multidrug resistance genes such as aminoglycoside adenyl transferase, streptothiricin- and hygromycin-resistant genes. In addition, the pCCDM183 plasmid sequence also contained genes for kanamycin kinase and spectinomycin o-adenyl transferase. The presence of aminoglycoside resistance genes in *Campylobacter* strains was also reported in previous studies (Tenover et al., 1992; Chen et al., 2013). Megaplasmids of more than 80 kb are present in the pTet plasmids (type-1) group (Marasini and Fakhr, 2016a,c; Miller et al., 2016). In addition to the common genes among pTet plasmids (Table 3), the megaplasmids were shown to have an inserted segment of bacteriophage genes or other types of mobile genetic components (Gunther et al., 2016; Marasini and Fakhr, 2016c). Some of these megaplasmids also contained the complete Type VI secretion system with all 14 core genes present (Marasini and Fakhr, 2016a). The fact that 14/29 plasmids sequenced in our laboratory were pTet plasmids (Table 2) is not surprising since we previously reported that tetracycline resistance was prevalent among *Campylobacter jejuni* and *Campylobacter coli* strains isolated from various retail meats (Noormohamed and Fakhr, 2012, 2013, 2014). Functional analysis of the virulence and antimicrobial resistance genes present on these plasmids is worth investigating and may shed some light on the role of these genes in conferring the corresponding phenotypes.

The type-2 plasmids, which ranged in size from 24 kb to 32 kb, were similar to few others deposited in the GenBank. This plasmid group primarily consists of plasmids from *C. coli* strains. However*, two* plasmids from *C. jejuni* strains are also found to be allied in this group (Figure 6). A plasmid present in *Helicobacter pullorum* (i.e., plasmid 229336_12) is a close ally of the type-2 plasmids (Figure 6). Most of the predicted genes in these plasmids are common to all plasmids in this group (type-2) except for a few hypothetical proteins. The core genome for type-2 plasmids included genes for conjugative transfer along with type IV secretion system genes such as virD4 and virB1(Table 4). Most of the conjugative transfer genes present in these plasmids were different from the Type IV conjugative transfer genes in other *C. jejuni* and *C. coli* plasmids (type-1 and type-3). The similarity between type-2 plasmids from *C. jejuni* and *C. coli* strains and the 229336_12 plasmid (*H. pullorum*) might indicate a possible route of transmission and genetic interchangeability of these plasmids between species. The pCCDM67S plasmid from our study is similar to a pVir type plasmid, previously studied by Bacon et al. (2002). The pCCDM67S plasmid also contains orthologs of the Type IV secretion system found in *Helicobacter pylori* (Bacon et al., 2000).

In addition to types 1-3, we characterized other small cryptic plasmids (type-4) that share similar genomic composition and arrangements with previously-characterized plasmids of diverse sources (Jesse et al., 2006; Miller et al., 2007). Most of these plasmids contain the replication initiator protein and some unknown hypothetical proteins. Some of these plasmids also contain genes coding for Mob proteins.

The phylogenetic analysis clearly shows numerous, well-resolved lineages comprised of complete sequences for all *Campylobacter* plasmids reported to date (Figure 6, Supplementary Figure S1). The branching pattern of the phylogenetic trees supports our categorization of *Campylobacter* plasmids according to pangenomic analysis for type 1, 2, and 3 plasmids. In MP tree, the most prevalent pTet plasmids (type-1) were all grouped in one lineage. Similarly, type-2 and type-3 (pVir) plasmids formed distinctive clades with similar plasmids from Genbank. Type 1 (pTet), type-2, and type-3 (pVir) plasmid groups consist of plasmid sequences from *C. jejuni/C. coli* strains. The small (<6 kb) plasmid sequences from *C. jejuni/C. coli* strains cluster with plasmids from other *Campylobacter* species. One plasmid cluster including plasmid mp1 (CP014569.1) consists of plasmids from mostly *C. fetus* and non-jejuni/coli species of *Campylobacter*. This cluster is found near type-2 plasmids. Since we have very little knowledge of the distribution of these *Campylobacter* plasmids, we used Maximum Parsimony for the analysis of the plasmid sequences. In a previous study done by (Crespo et al., 2016), similar types of phylogenetic relationships were observed where the majority of plasmids with *tetO* genes were allied in one cluster and some smaller plasmids were allied in another cluster. Results of NeighborNet analysis indicate that plasmids found in *Campylobacter* species likely have a convoluted evolutionary history (Supplementary Figure S1). Nonethless, there is a 1:1 correspondence between the network (Supplementary Figure S1) and the tree from MP analysis (Figure 6) regarding major groupings of type 1, type 2 and type 3 plasmids (Supplementary Figures S1B,D,F). The root also shows correspondence between the two analyses. A portion of the type 4 group (Figure 6) forms a loose cluster in the network analysis (Supplementary Figure S1E). The type 4 group is a non-monophyletic assemblage in the MP analysis, too. This indicates that type 4 is either the most diverse plasmid type or may be comprised of additional, unrecognized types. We can simply state that the well-resolved portions of the phylogeny provide a reasonable inference for relationships among plasmids.

Most of the isolated and sequenced plasmids are associated with isolates of *C. jejuni* and *C. coli*. This observation is consistent with the fact that most clinical cases of *Campylobacter* infection are associated with *C. jejuni* and *C. coli* strains (Acheson and Allos, 2001; Gillespie et al., 2002). The absence of any species-specific plasmid clades indicates intra-species dissemination of plasmids among *Campylobacter* species. Several divergent lineages are present in our analyses (Figure 6, Supplementary Figure S1) and these may be representatives of larger, but under-sampled plasmid lineages. Thus, the results of this study indicate that additional sampling will be needed to more fully understand the evolution and transmission of *Campylobacter* plasmids.

## Author contributions

MF conceived the research idea and design. DM and AK performed the experimental procedures. DM, AK, and MB performed the phylogenetic analysis. DM, AK, MB, and MF prepared the manuscript.

### Conflict of interest statement

The authors declare that the research was conducted in the absence of any commercial or financial relationships that could be construed as a potential conflict of interest.
