# Supplementary material for: Phylogenetic Relatedness Among Plasmids Harbored by Campylobacter jejuni and Campylobacter coli Isolated From Retail Meats
Source: Front Microbiol. 2018 Sep 12;9:2167. doi: 10.3389/fmicb.2018.02167 (PMC6145009; doi:10.3389/fmicb.2018.02167)
Supplement: Supplementary file 10 [file Image_1.pdf]

Supplementary Figure S1: NeighborNet analysis among all plasmids from *Campylobacter* species available in GenBank. **S1a:** Neighbor-net among all plasmids of *Campylobacter* species {shaded colors represent types of plasmid according to pangenome analysis (**Fig. 1**) and MP tree (**Fig. 6**)}. **Fig. S1b, S1c, S1d, S1e, S1f, S1g** and **S1h** represent major groupings of plasmids found in neighbor-net analysis which corresponds with MP tree **(Fig. 6)**.

Fig. S1a

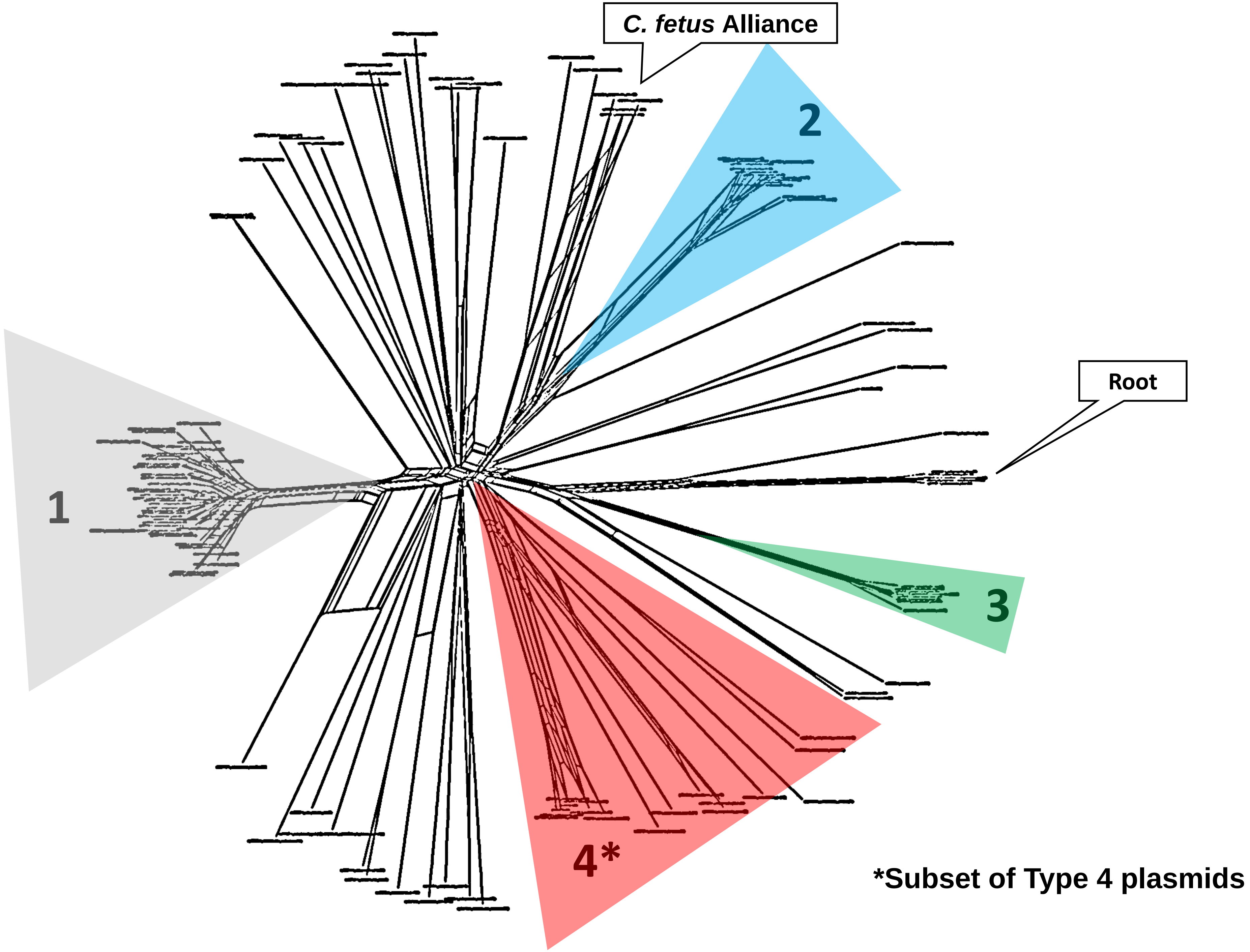

Fig. S1b

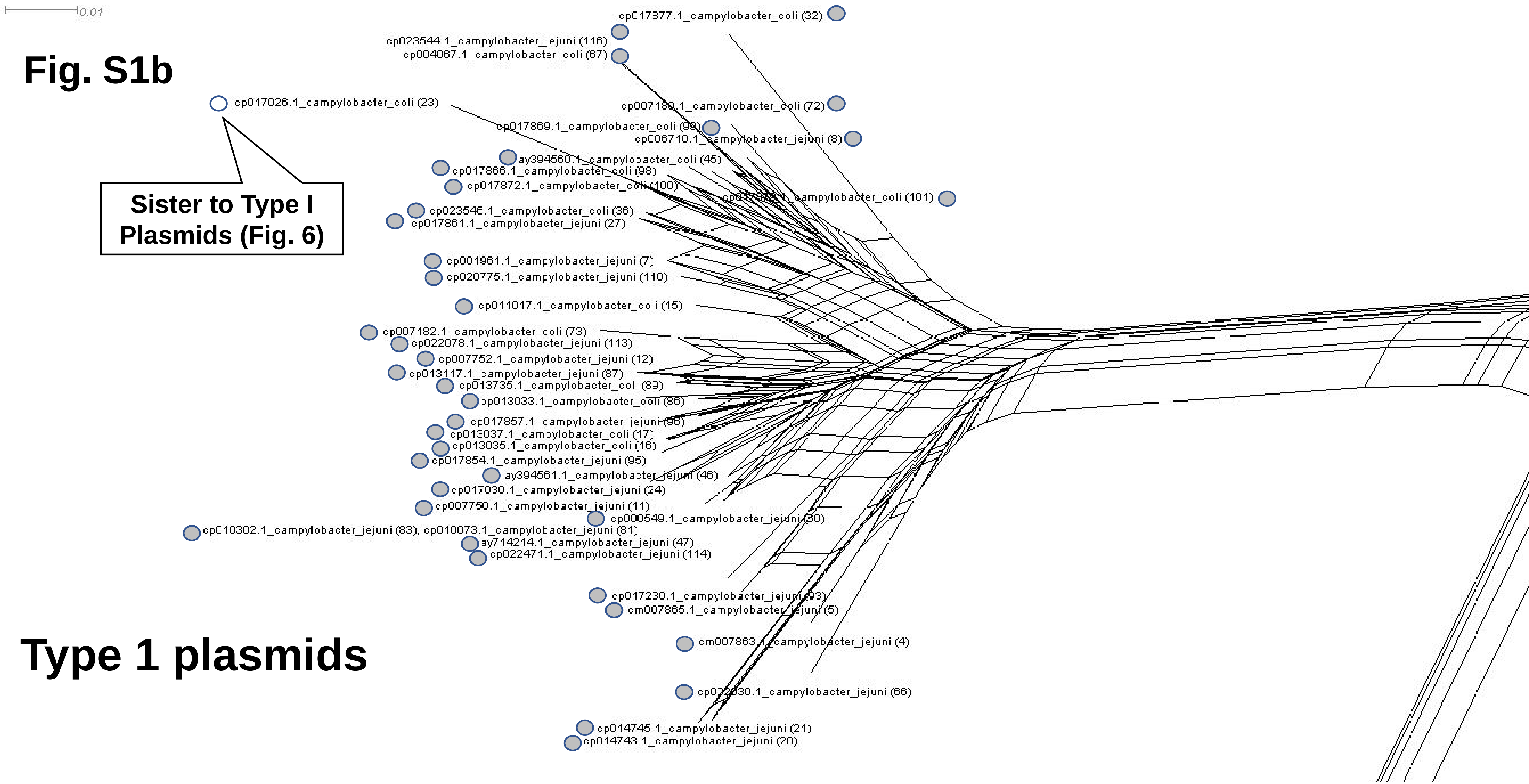

Type 1 plasmids

Fig. S1c

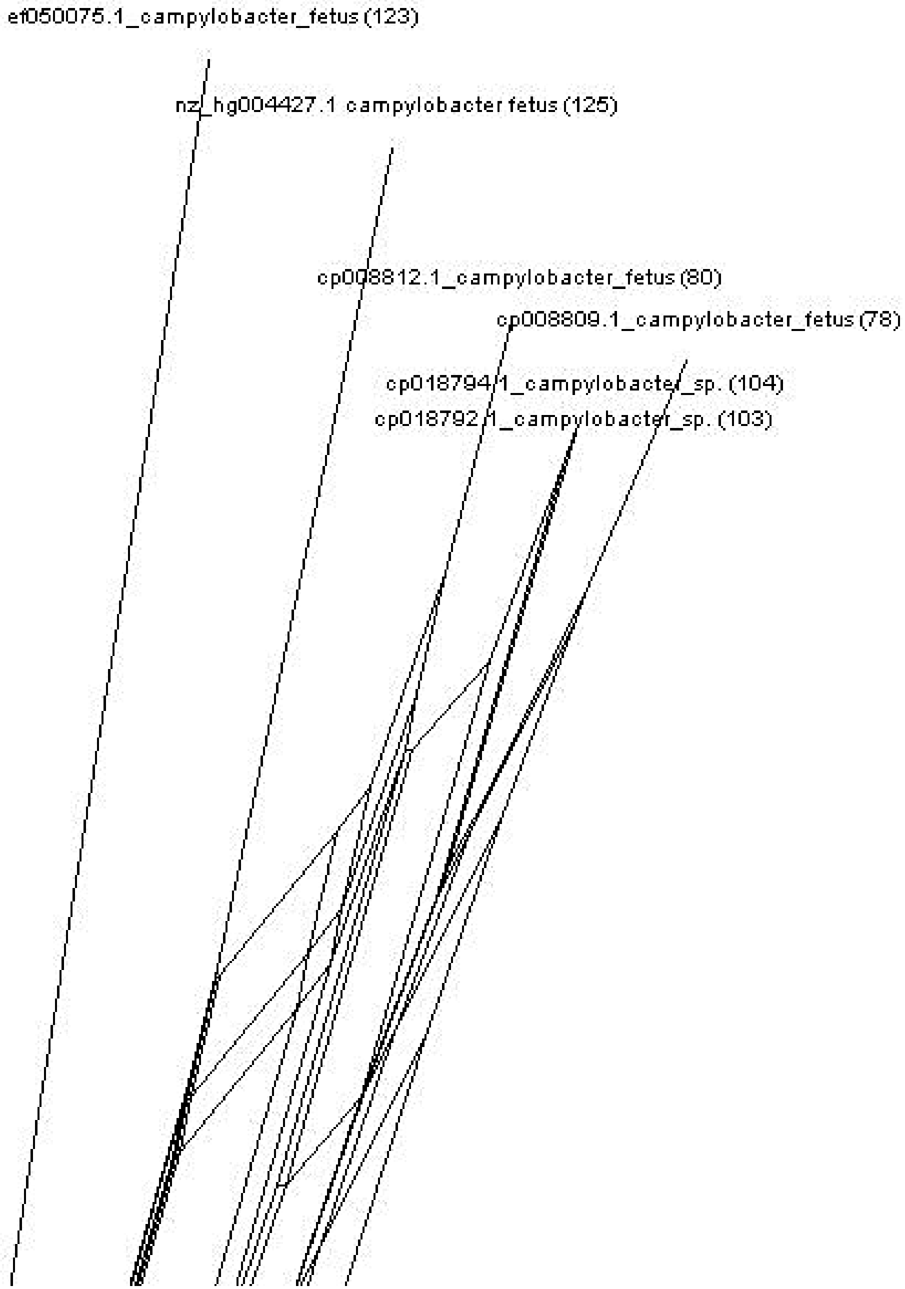

C. fetus Alliance

Fig. S1d

Type 2 plasmids

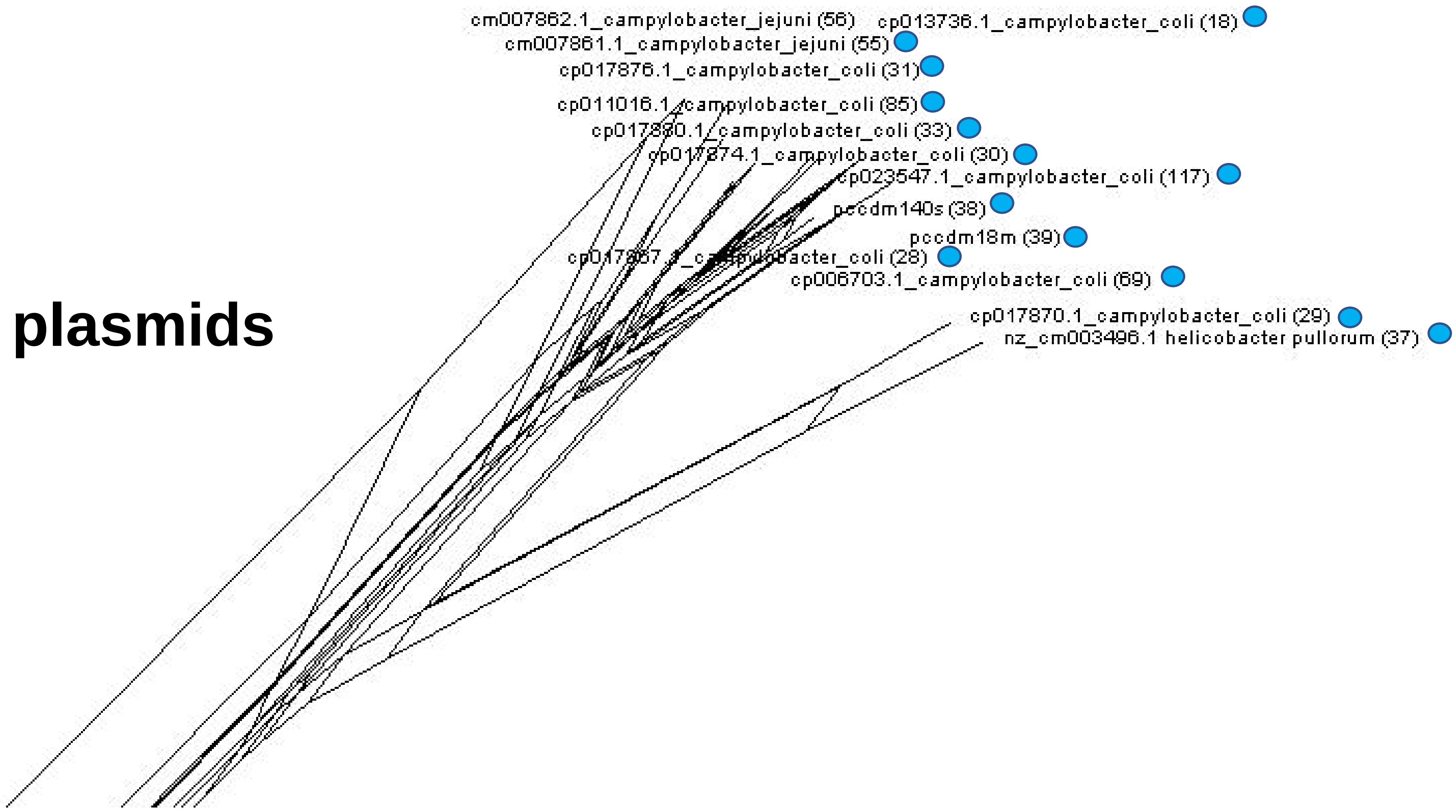

Fig. S1e

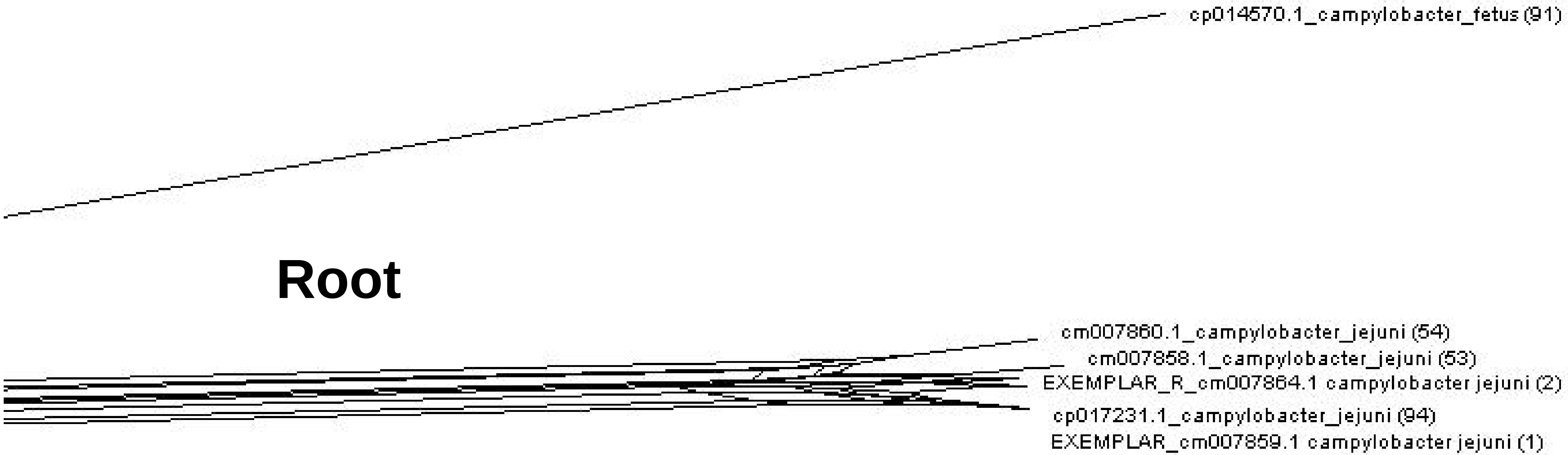
cp014570.1_campylobacter_fetus (91)
Root
cm007860.1_campylobacter_jejuni (54)
cm007858.1_campylobacter_jejuni (53)
EXEMPLAR_R_cm007864.1 campylobacter jejuni (2)
cp017231.1_campylobacter_jejuni (94)
EXEMPLAR_cm007859.1 campylobacter jejuni (1)

Fig. S1f

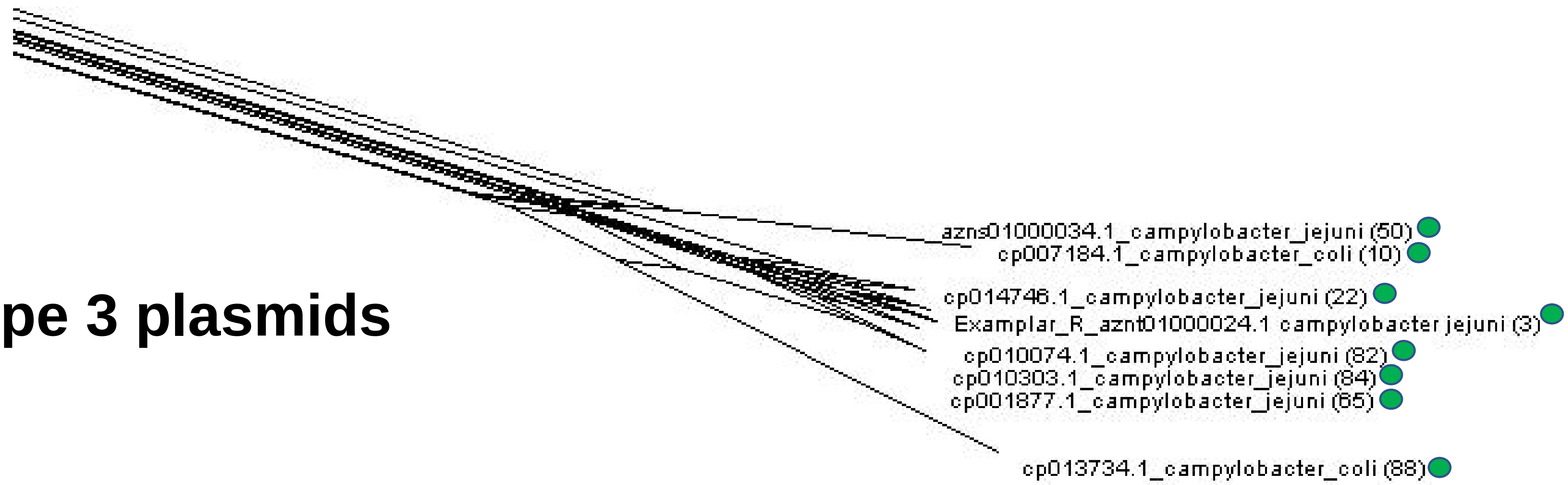

Type 3 plasmids

Fig. S1g

Type 4 plasmids (subset 1)

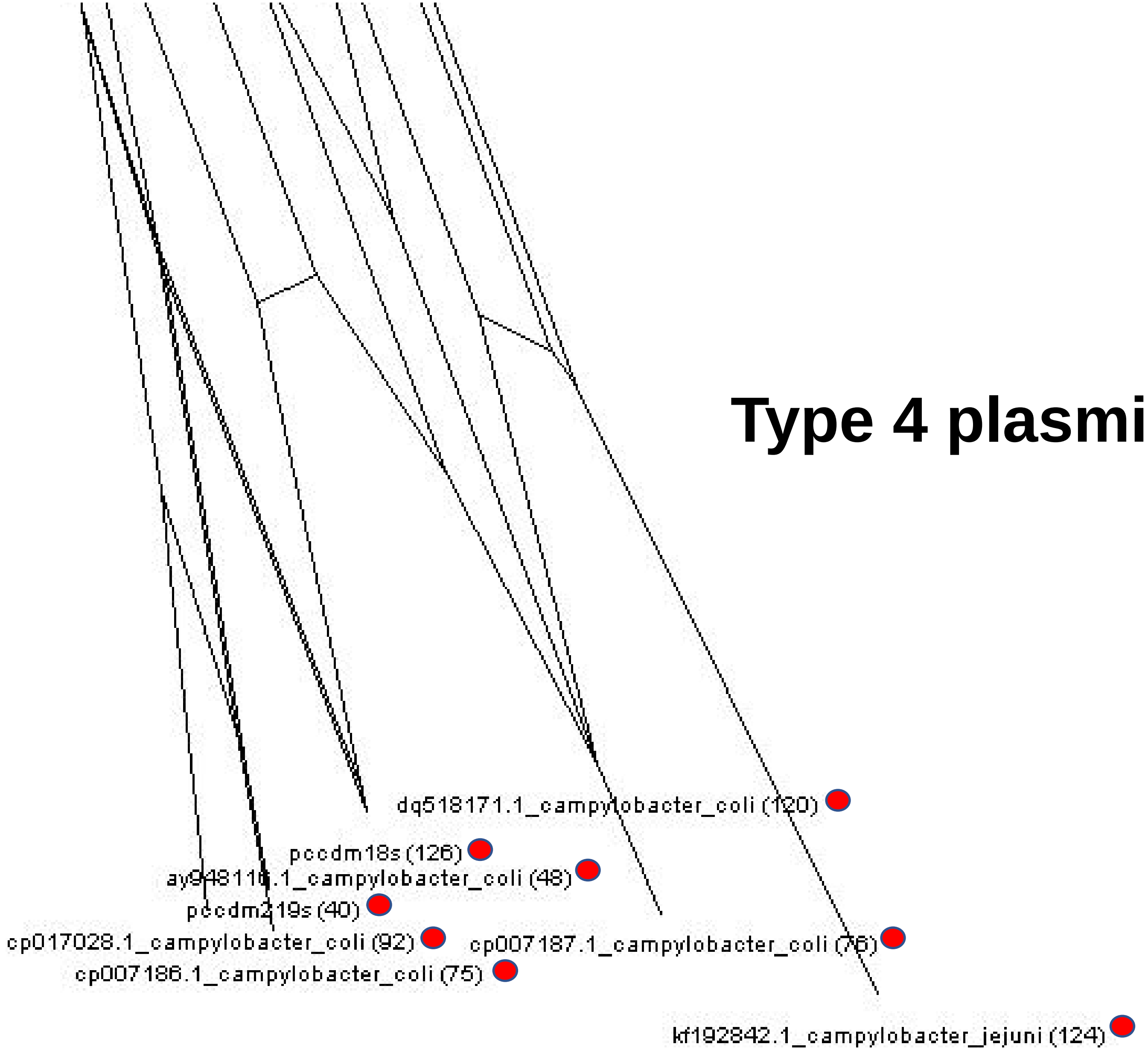

Fig. S1h

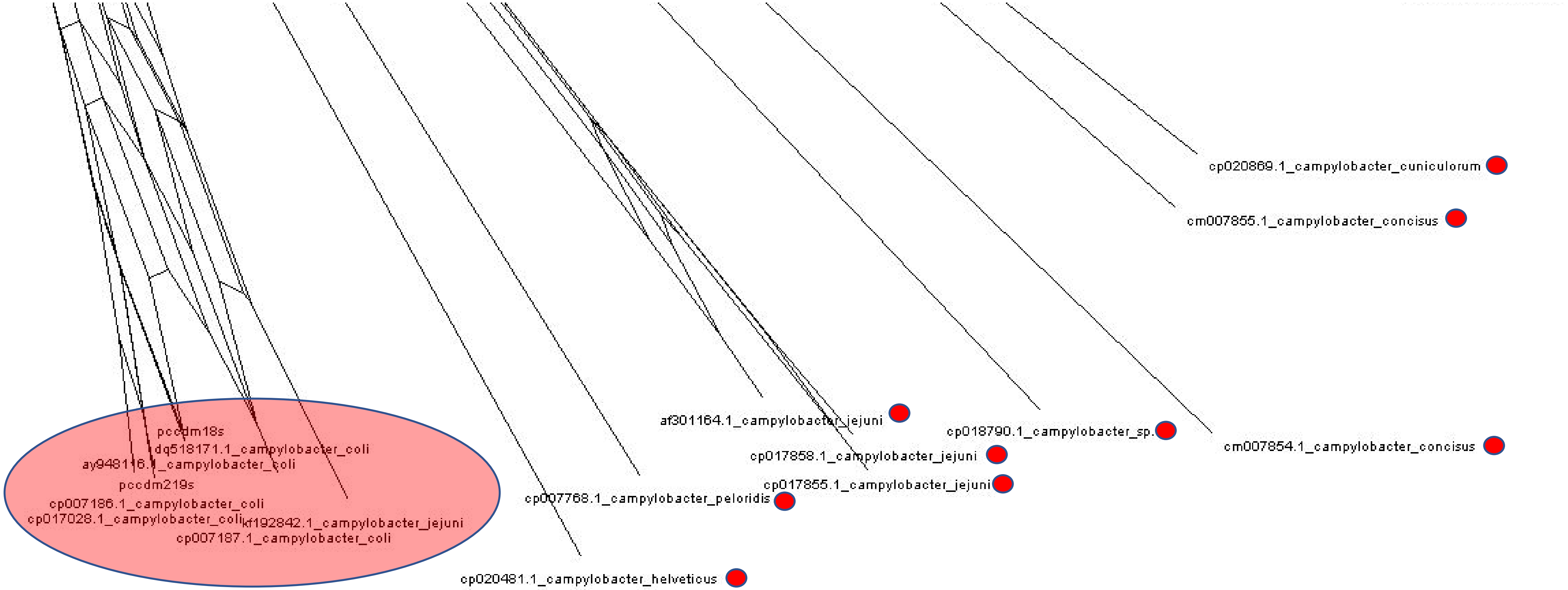

Type 4 plasmids (subset 2)
